# Optimal Sparsity Selection Based on an Information Criterion for Accurate Gene Regulatory Network Inference

**DOI:** 10.3389/fgene.2022.855770

**Published:** 2022-07-13

**Authors:** Deniz Seçilmiş, Sven Nelander, Erik L. L. Sonnhammer

**Affiliations:** ^1^ Department of Biochemistry and Biophysics, Science for Life Laboratory, Stockholm University, Solna, Sweden; ^2^ Science for Life Laboratory, Department of Immunology, Genetics and Pathology, Uppsala University, Uppsala, Sweden

**Keywords:** sparsity selection, information criteria, gene regulatory network inference, gene expression data, noise in gene expression

## Abstract

Accurate inference of gene regulatory networks (GRNs) is important to unravel unknown regulatory mechanisms and processes, which can lead to the identification of treatment targets for genetic diseases. A variety of GRN inference methods have been proposed that, under suitable data conditions, perform well in benchmarks that consider the entire spectrum of false-positives and -negatives. However, it is very challenging to predict which single network sparsity gives the most accurate GRN. Lacking criteria for sparsity selection, a simplistic solution is to pick the GRN that has a certain number of links per gene, which is guessed to be reasonable. However, this does not guarantee finding the GRN that has the correct sparsity or is the most accurate one. In this study, we provide a general approach for identifying the most accurate and sparsity-wise relevant GRN within the entire space of possible GRNs. The algorithm, called SPA, applies a “GRN information criterion” (GRNIC) that is inspired by two commonly used model selection criteria, Akaike and Bayesian Information Criterion (AIC and BIC) but adapted to GRN inference. The results show that the approach can, in most cases, find the GRN whose sparsity is close to the true sparsity and close to as accurate as possible with the given GRN inference method and data. The datasets and source code can be found at https://bitbucket.org/sonnhammergrni/spa/.

## Introduction

Genes are responsible for orchestrating the biochemical processes in a living organism, which is only possible through a well-organized system of gene regulatory interactions called a gene regulatory network (GRN). An alteration of the system may result in complex genetic diseases, and potential treatment targets for these diseases can be identified by inferring reliable GRNs as they can reveal important mechanisms in the underlying system.

Despite the importance of an accurate GRN inference, it has been difficult to achieve due to several data-related issues such as biases and noise (Tjärnberg et al., 2015; Tjärnberg et al., 2017). The application of preprocessing approaches to noisy datasets followed by GRN inference through an accurate method has been shown on *in silico* data to overcome the noise-related obstacles in the inference to a degree (Seçilmiş et al., 2020; Seçilmiş et al., 2021), when the accuracy of the GRN inference can be measured with a known true network which is available for synthetically generated data. The accuracy is most commonly measured by the area under the precision-recall and receiver-operating characteristic curves (AUPR and AUROC, respectively) (Huynh-Thu et al., 2010; Madar et al., 2010; Marbach et al., 2012; Bellot et al., 2015), that consider the entire range of sparsities, from an empty to a full network.

For practical purposes, however, it is important to be able to infer the single best GRN, which should be as close to the true GRN as possible. In a benchmark with simulated data from a known true network, this can be assessed by accuracy measures such as the F1-score. However, in the absence of a known true network when using real biological datasets where underlying novel genetic interactions are yet to be identified as potential treatment targets, none of these measurements can be used to evaluate the accuracy of the inferred GRNs. In such situations, the selection of the best GRN is of critical importance and most often made by an arbitrary cut-off on the sparsity, which is usually ∼1–3 three links per gene on average for biological reasons (Martínez-Antonio et al., 2008; Seçilmiş et al., 2020). However, this approach does not guarantee the selected GRN to represent the best and most optimal model within the space of all possibilities in terms of both accuracy and the information content. Previous attempts have been published, for instance, Tjärnberg et al. (2013) proposed a method to reconstruct the gene expression from a set of inferred GRNs whose sparsity ranges from full to empty, and showed that this approach works well for informative data but not when the noise level is high. Morgan et al. (2020) proposed a method for assessing GRN quality based on cross-validated fitting of the GRN’s topology to expression data which was applied to select an optimal GRN for a biological dataset.

Methods such as LASSO (Tibshirani, 1996; Friedman et al., 2010) use a regularization approach through an internal penalty term (called the L1-norm) to obtain a sparse GRN. However, they do not offer any guidance on what value to set the L1 penalty to find the optimal sparsity. To this end, one could potentially use the Akaike Information Criterion (AIC) or Bayesian Information Criterion (BIC), since these approaches would, in principle, minimize the information loss with the minimum required number of independent variables across all given models. These approaches have previously been used in combination with penalty-based GRN inference approaches (Menéndez et al., 2010), such as the graphical LASSO (Friedman et al., 2008). However, it failed for AIC and is also not applicable to non-penalty-based GRN inference methods such as GENIE3 (Huynh-Thu et al., 2010).

Here, we present SPA, a sparsity selection algorithm that is inspired by the AIC and BIC in terms of introducing a penalty term to the goodness of fit, but is developed particularly for GRN inference to identify the most mathematically optimal and accurate GRN within a set of GRNs from varying sparsities inferred by any inference method. The main idea behind the algorithm is to determine the optimal model in which regulator genes are alone capable of explaining target genes with minimum information loss, given the gene expression data and its perturbation design.

## Methods

### SPA: The Sparsity Selection Algorithm

**FIGURE 1:**
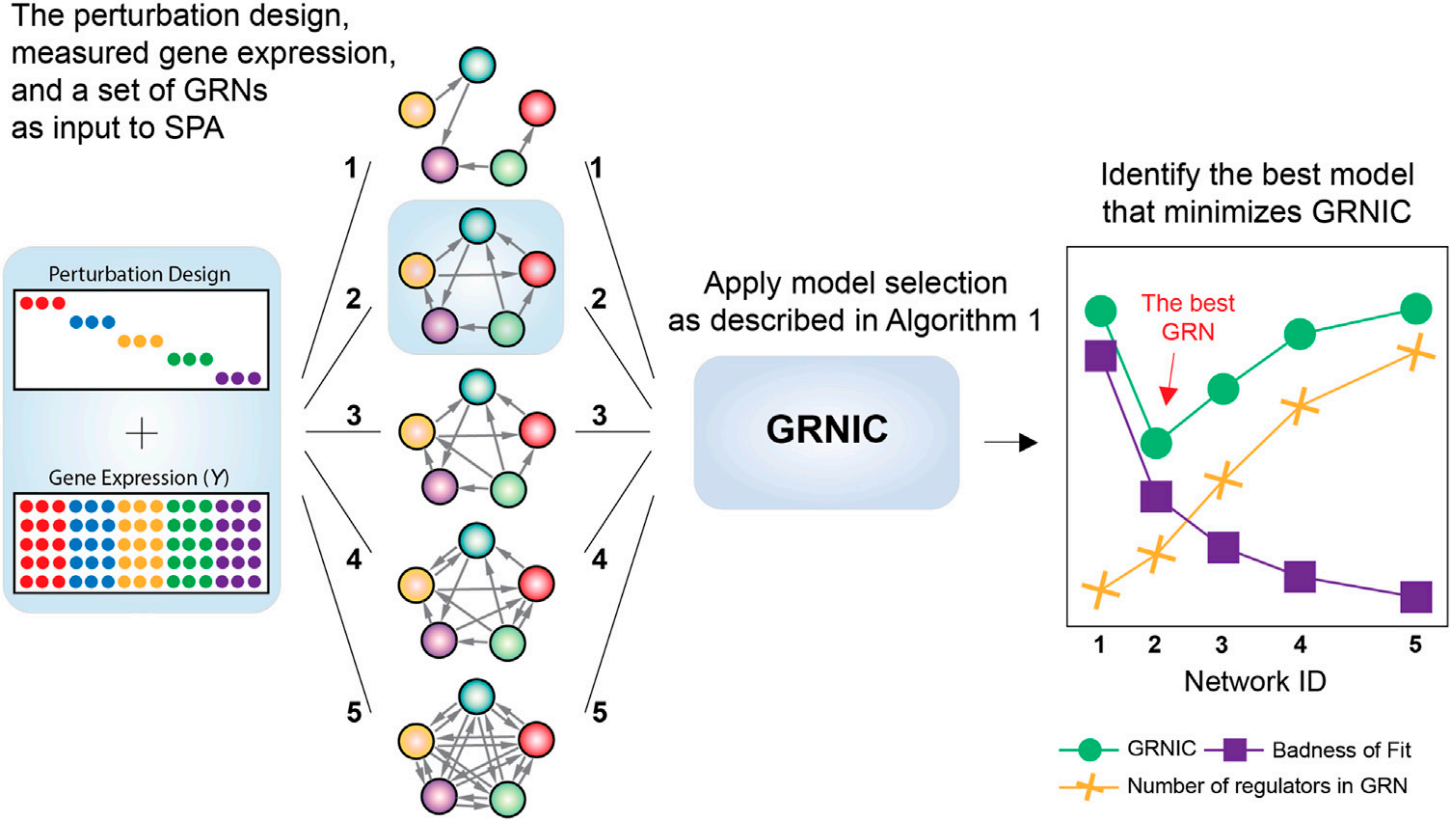
Workflow of SPA. SPA takes a set of inferred GRNs with varying sparsities, the measured gene expression in fold changes, and the perturbation design as input. It then uses the GRN Information Criterion (GRNIC) as described in Algorithm 1 and identifies the GRN that minimizes GRNIC as the best GRN.

SPA is a model selection pipeline that takes as input *S* GRNs with different sparsities inferred by any inference method, gene expression measured, and the perturbation design. It then assesses the quality of each input GRN *i (1, … , S)* based on its information content as detailed in Algorithm 1, and identifies the model minimizing GRNIC as the best GRN (Figure 1).

Algorithm 1

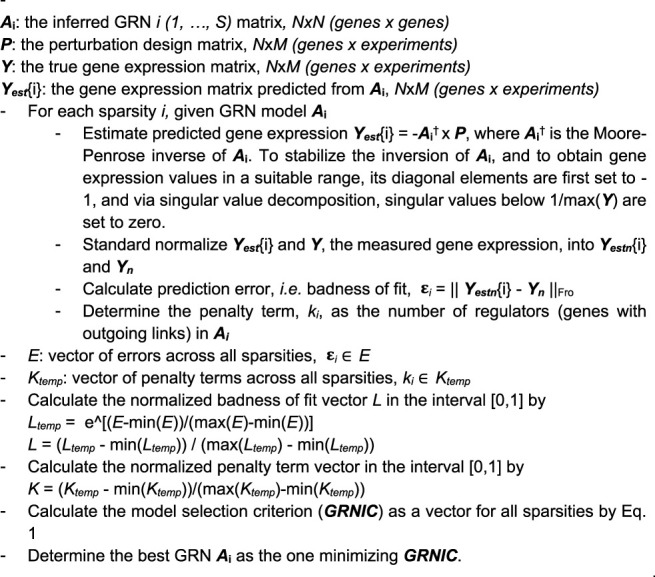

The assessment of a GRN model’s information content is made by an information criterion inspired by AIC and BIC, called GRNIC from GRN Information Criterion, and calculated according to Eq. 1. This criterion aims to balance the error in predicting the underlying gene expression (badness of fit) and the number of variables (regulators) in the model. Therefore, the GRN that minimizes GRNIC is expected to include a set of variables which alone are sufficient enough to reconstruct the measured gene expression, without needing more variables.

GRNIC=K+L. (1)

In Eq. 1, K refers to the normalized penalty term, here set to the number of genes that regulate at least one other gene in the GRN, and L denotes the normalized badness of fit, here based on the prediction errors of the estimated gene expression from the GRN, calculated as described in Algorithm 1. The model for predicting the gene expression from a GRN i as -***A***_**i**_^†^ x ***P*** is derived by Tjärnberg et al. (2015). It is preceded with a conditional step of removing singular values below 1/max(***Y***) that is almost never used but is included as a safeguard against unstable inversions.

The normalization step to the terms of GRNIC was added as their units do not naturally relate to each other, making them incomparable without it. Finding the minimum GRNIC rewards low badness of fit (high goodness of fit) considering a penalty that increases with the number of variables. To implement GRNIC as close to AIC as possible, we here took *e* to the power of the badness of fit as an opposite equivalent of taking the natural logarithm of the goodness of fit.

### GRN Inference

We applied two different types of approaches: non-penalty-based and penalty-based methods. As the non-penalty-based method we chose GENIE3, and for the penalty-based methods, we chose LASSO and Ridge regression. The code for the used methods is available at https://bitbucket.org/sonnhammergrni/genespider.

GENIE3 was run with the following parameter settings: number of regulators: all genes; tree method: random forests; and number of trees: 1,000. Note that reverse edge direction is used because it is considerably more accurate. This resulted in a fully connected GRN with directed interactions that all have positive weights. All GRNs were then extracted whose sparsity ranged from 1–5 interactions per gene on average. For a 100-gene dataset, this corresponds to 401 different sparsities. The rationale behind this is that a biologically relevant GRN would contain ∼1–3 interactions per gene on average (Martínez-Antonio et al., 2008; Marbach et al., 2012).

LASSO was run as described by Tjärnberg et al. (2015) using the glmnet Matlab package with alpha = 1, and GRNs were inferred with as many sparsities as can be obtained given the data. This was followed by extracting the GRNs whose sparsity ranges from 1–5 interactions per gene on average, following the same aforementioned biological motivation.

Ridge regression was also run using the glmnet Matlab package with alpha = 0. Different sparsities were obtained by applying cutoffs to the full GRN that Ridge regression outputs.

### Data

Five 100-gene subnetworks were extracted from the complete *E. coli* GRN available in the GeneNetWeaver network and data generation tool (Schaffter et al., 2011) to be used as the “true” GRNs (in other words, the underlying regulatory system, where the topological properties of the *E. coli* network are preserved), for gene expression data generation. Autoregulatory interactions (self-loops) exist in these true GRNs for the system’s stability, but none of them was used later on when measuring inference accuracy, to make it solely determined by non-self-loops. To maximize the regulatory effect in subsets, all genes were requested to be a transcription factor, yet only a fraction of all genes had regulatory effects: 0.53, 0.52, 0.53, 0.50, and 0.53 for the five true GRNs. The vertices (genes) were drawn randomly with the “greedy” edge selection (a GeneNetWeaver network extraction setting). The sparsity of the extracted true GRNs is defined as the number of interactions per gene on average, and ranges between 1.5 and 1.95 excluding self-loops. For each true subnetwork, noise-free steady-state single knockdown gene expression data were generated from ordinary differential equations (a data generation setting in GeneNetWeaver). Fold changes in gene expression following the system’s perturbations were calculated as log base two of the ratio between experiment and wild-type expression. The gene expression data created by GeneNetWeaver are an NxN matrix ***Y*** (*N* = 100) of single replicate experiments, which places the perturbation indications as −1 on the diagonal in the experiment design matrix ***P***. Then, concatenating these matrices three times with themselves yields a three-replicate matrix of size Nx3N. We separately generated the corresponding Gaussian noise matrices with two different signal-to-noise ratios (SNRs) corresponding to “high” and “low” noise levels from Supplementary Equation S1 and added these to the ***Y*** matrix.

## Results

**FIGURE 2:**
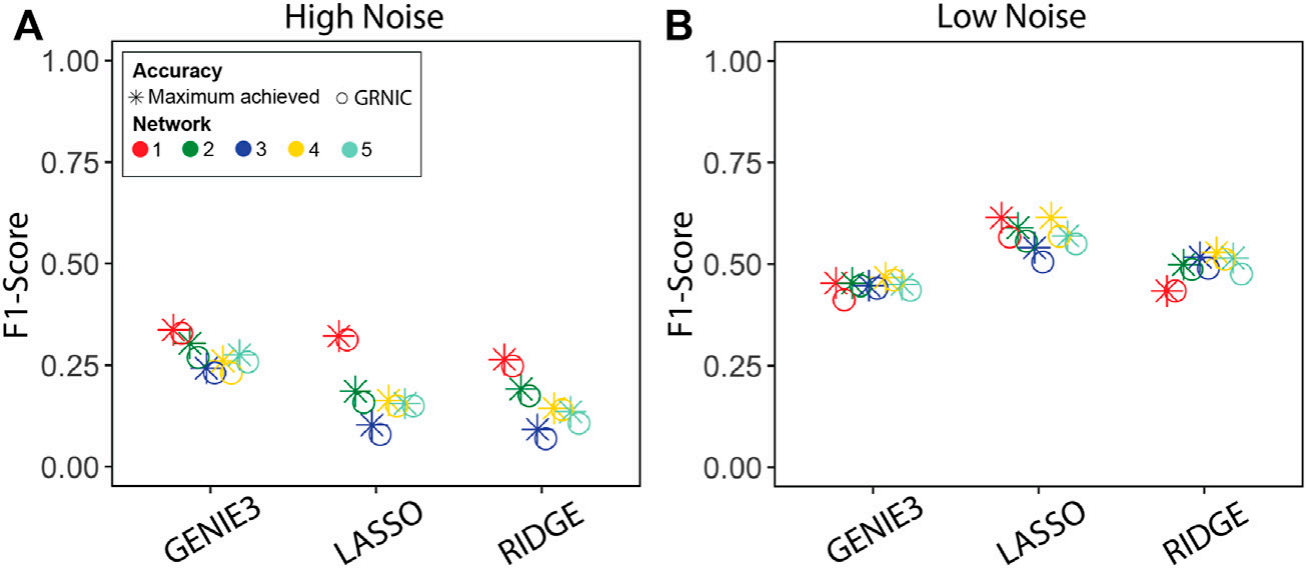
Performance evaluation of the sparsity selection pipeline in terms of the F1-score. F1-scores of the inferred GRNs from datasets generated by GeneNetWeaver with **(A)** high and **(B)** low noise levels. Each panel contains F1-scores from five datasets for two categories: GRNIC (circle) and maximum achieved in inference (star).

**FIGURE 3:**
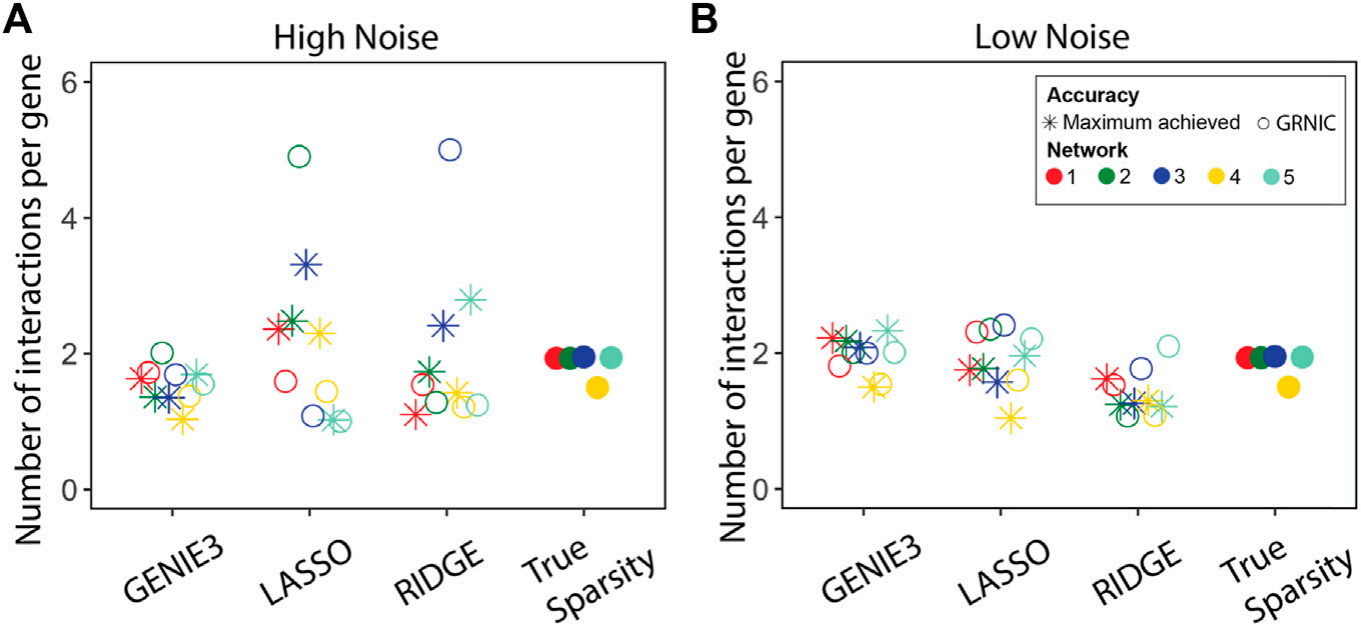
Performance evaluation of the sparsity selection pipeline in terms of sparsity. The sparsity of the inferred GRNs (the GRN having the maximum F1-score, and the one selected by GRNIC) is shown in terms of the average number of links for **(A)** high and **(B)** low noise levels for the five networks. The sparsities of the five true GRNs are shown in an extra column to the right.

We performed GRN inference using GENIE3, LASSO, and Ridge regression on synthetic datasets and measured the GRN inference accuracy in terms of the F1-score. Given a set of GRNs of different sparsities for each method, we applied GRNIC model selection criterion as described in the Methods section (Algorithm 1; Eq. 1). F1-scores of the selected GRNs were then compared with the maximum F1-score obtained from the investigated range (Figure 2). In addition to this, we evaluated the sparsities of the selected GRNs (Figure 3).

The results show that the maximum accuracy of the GRN inference method can be nearly achieved by SPA using GRNIC, for all applied inference methods. In particular, we observed a noise-related trend in the accuracy in terms of the F1-score, where the GRN inference accuracy increased relative to the decreasing noise, most notably for LASSO and Ridge regression, from “high” to “low” noise. SPA was able to identify GRNs very close to the maximum accuracy GRNs for all methods in most datasets. There was a slight deviation from the maximum achieved F1-score for network 1 for GENIE3, networks 1 and 4 for LASSO, and network 5 for Ridge regression, at the low noise level. At the high noise level, SPA was able to identify GRNs whose F1-scores are almost identical to the maximum achieved for all methods.

We analyzed the two terms of the GRN information criterion (GRNIC) from Eq. 1, that is, the penalty term (*K*) and badness of fit term (*L*), separately, and assessed their effect on GRNIC (Supplementary Figures S1–S5). We observed that in most cases, the two terms of GRNIC behave as expected, where the badness of fit decreases relative to the increasing number of regulators in the model (see e.g. Supplementary Figure S1A). However, there are a few cases that do not behave as expected, which we investigate as follows.

### Issue of GRNIC Curve Not Finding a Minimum at the True Sparsity

We observed a few cases where, even though both the penalty term (*K*) and the badness of fit term (*L*) behave as expected, the resulting GRNIC values are not minimized around the true sparsity, and instead peak here (see e.g. Supplementary Figure S2E). This situation can occur if the increase in the number of regulators goes faster than the decrease in the badness of fit, causing the penalty term to dominate the badness of fit when calculating GRNIC. There is no obvious solution to this issue, but either an improved function that better captures the badness of fit or an adjustment to the penalty term *K* could potentially resolve it.

### Issue of Aberrant Badness of Fit

The badness of fit is expected to decrease with the addition of regulators, that is, going from sparser GRNs to denser ones, because of the increasing number of variables. However, a very clear parabolic curve is formed by the badness of fit values from the GENIE3 GRNs at the high noise level dataset generated from network 4 (Supplementary Figure S4A) and less clearly from network 3 (Supplementary Figure S3A). This type of behavior, however, does not prevent SPA from finding the optimal sparsity. Another concerning behavior of the badness of fit was observed for Ridge regression GRNs at the low noise level, especially for networks 3–5, where the badness of fit increases relative to the increasing number of regulators (Supplementary Figures S2F, S4F), which is the opposite of what is expected. We have not found any clear explanation as to why these two situations occur. In both types of aberrant behavior, the increased badness of fit with increased GRN density indicates that the larger models incorporate links that are less predictive, for instance, false positives that have a strong negative impact on the goodness of fit.

To explore how the badness of fit curves compare to what is expected by chance, we applied an experiment-wise random shuffling to the normalized gene expression matrices reconstructed by the GRN inference methods. Both badness of fit curves, the actual and shuffled, are visualized together in Supplementary Figures. S6–S10. The trend observed in most of the actual badness of fit curves, that is, gradually decreasing badness of fit with decreasing sparsity, was lost for the shuffled curves, which also had a very stochastic behavior. This adds further support to the validity of the applied badness of fit for the purpose of assessing the ability of a GRN to reconstruct the underlying gene expression.

Despite a few aberrant cases, the GRNs identified by SPA are almost as accurate as of the maximum achieved accuracy. To further compare the sparsities of the GRNs identified by SPA with those that achieved the maximum accuracy levels among others, we calculated the number of interactions per gene on average (Figure 3).

For GENIE3, SPA selected GRNs closer to the true sparsity than the other methods, and for most networks, it also came closer than the most accurate sparsity. This suggests that while GENIE3 predictions are not optimal, the criteria applied by SPA find the set of regulators that are optimal to reconstruct the underlying gene expression at both noise levels. The situation for LASSO and Ridge regression is not as promising as observed for GENIE3 at the high noise level. Some sparsities were overestimated while some others were underestimated compared with the true sparsity levels. However, the GRNs that achieved the maximum F1-scores also deviated from the true sparsity levels, suggesting that at this noise level, it is a difficult task to accurately reconstruct GRNs from the underlying data. This hypothesis is supported by the sparsity comparison at the low noise level, where both SPA GRNs and those which achieved the maximum F1-scores have similar sparsities to the true levels. In some cases, for example, for networks 3 and 5 at low noise levels, sparsities of the GRNs identified by SPA are closer to the true sparsity levels than those which achieved the maximum F1-scores. This means, for some cases, SPA is able to eliminate malefic interactions/regulators without sacrificing a significant portion of accuracy.

## Discussion

The ability of SPA in identifying a GRN that approaches the maximum achieved accuracy with a biologically relevant sparsity solves an old and vexing problem in the field. It can provide guidance for selecting the most optimal and accurate GRN from a set of inferred ones, which is important, for instance, when the ultimate goal is to determine novel treatment targets for underlying genetic diseases from biological data in the absence of a true GRN.

There are a few obstacles to doing this, of which the most important one is noise in gene expression. This study shows that, even though SPA identifies the most accurate GRN, its accuracy may still not be good enough for a biological discovery if the noise levels are high, referring to possibly unreliable predictions. Therefore, when using SPA, one should always note that the highest possible accuracy that SPA can achieve is only limited to the applied GRN inference method’s ability to compensate for the noise.

SPA relies on the prediction accuracy of a set of input GRNs in reconstructing the underlying gene expression, given the perturbation design. Therefore, its usage is, to some extent, limited to those methods inferring signed GRNs where not only the direction but also the sign of the interaction, that is, whether activation or inhibition, is known, if one wants to ensure mathematical suitability. However, our application to GRNs inferred by GENIE3 showed that SPA can overcome this limitation and still identify an accurate GRN at a reasonable sparsity. It would be possible to further extend its application to undirected GRNs (Faith et al., 2007; de Matos Simoes and Emmert-Streib, 2012) to see to what degree SPA can find the most optimal GRN in such cases. However, we consider this problem out of the scope for this particular study since the main motivation behind identifying the most optimal GRN is to be able to understand the causality in gene regulation, and the most straightforward way of achieving this goal is to apply methods which are suitable for this purpose.

The replacement of the goodness of fit term by the prediction error in calculating the information-theoretical criteria required a few adjustments in their formulation, including a normalization step for the estimated gene expression data, and a scaling step to the badness of fit and number of variables to allow for a fair comparison between the two terms of the information criterion. This was necessary to allow for a comparison between predicted and measured gene expression since, depending on the magnitude of the GRN content and measured gene expression, the predicted gene expression can vary significantly, potentially confounding biases. A series of normalization steps on the predicted gene expression allowed both measured and predicted gene expression to be in the same range, therefore providing a more balanced comparison to the problem. The results support the methodology behind both the applied badness of fit calculation and the general formula of SPA.

The GRNIC algorithm includes a step in which the exponential of the badness of fit is calculated. This was carried out to implement GRNIC as close to AIC as possible, as an opposite equivalent of taking the natural logarithm of the goodness of fit, and also, we noticed that it improved performance. We also evaluated a number of alternative transformation functions such as square, cube, and logarithm of 1 minus the scaled badness of fit, to see which one performed the best. We concluded that even if some cases were improved with other functions, on the whole, there was no improvement and we, therefore, prefer to stay with the formulation closest to the original AIC. A potential future improvement could be to adapt the function to certain properties in the predicted and/or measured expression data. Because applying a transformation function can radically change the scale of the badness of fit, we apply normalization to ensure that it is in the same range as the penalty term.

In addition to the overall accuracy of SPA in identifying the most accurate GRNs near the true sparsity levels, we also focused on a few aberrant cases, some of which were possible to explain in terms of the negative effect of high noise levels, while some other questions raised by SPA remained unanswered. These may be answered by other researchers in the field together with what is present in this study, possibly inspiring an even better solution to the model selection problem in a larger context.

In conclusion, the implemented sparsity selection approach introduces a great advance to the field since achieving the highest possible accuracy is now made possible with the combination of a GRN inference method and SPA. We foresee that more novel gene regulatory interactions will be identified from the best possible GRNs using our algorithm, and potential treatment targets will be proposed.

## Data Availability

The datasets and source code can be found at https://bitbucket.org/sonnhammergrni/spa/.
