# Supplementary material for: Optimal Sparsity Selection Based on an Information Criterion for Accurate Gene Regulatory Network Inference
Source: Front Genet. 2022 Jul 13;13:855770. doi: 10.3389/fgene.2022.855770 (PMC9340570; doi:10.3389/fgene.2022.855770)
Supplement: Supplementary file 1 [file DataSheet1.PDF]

Supplementary material for "**Optimal sparsity selection based on an information criterion for accurate gene regulatory network inference**".

Deniz Seçilmiş[1], Sven Nelander[2], Erik L. L. Sonnhammer[1]*

[1]Department of Biochemistry and Biophysics, Stockholm University, Science for Life Laboratory, Box 1031, 17121 Solna, Sweden,
[2]Science for Life Laboratory, Department of Immunology, Genetics and Pathology, Uppsala University, Uppsala, Sweden

*To whom correspondence should be addressed: erik.sonnhammer@scilifelab.se

Supplementary Section: Noise generation for gene expression.
Noise is generated separately for each noise-free gene expression data, so that the noise in gene expression can be controlled by signal-to-noise ratio (SNR). For each given SNR: 0.01 and 0.1, respectively, for high and low noise levels, a standard deviation is calculated according to Suppl. Eq. S1 to be used to generate random Gaussian noise, which would result in the desired SNR when measured from the gene expression data.

$$\sigma_E = \frac{min(\gamma(\check{Y}))}{SNR_Y \sqrt{\chi^{-2}(\alpha, NM)}} \qquad \text{(S1)}$$

In Eq. S1, $\gamma(\check{Y})$ is the singular values from the singular value decomposition of the noise-free gene expression matrix, $\check{Y}$. Then random Gaussian values are generated from σ_E, and added to the noise-free gene expression data.

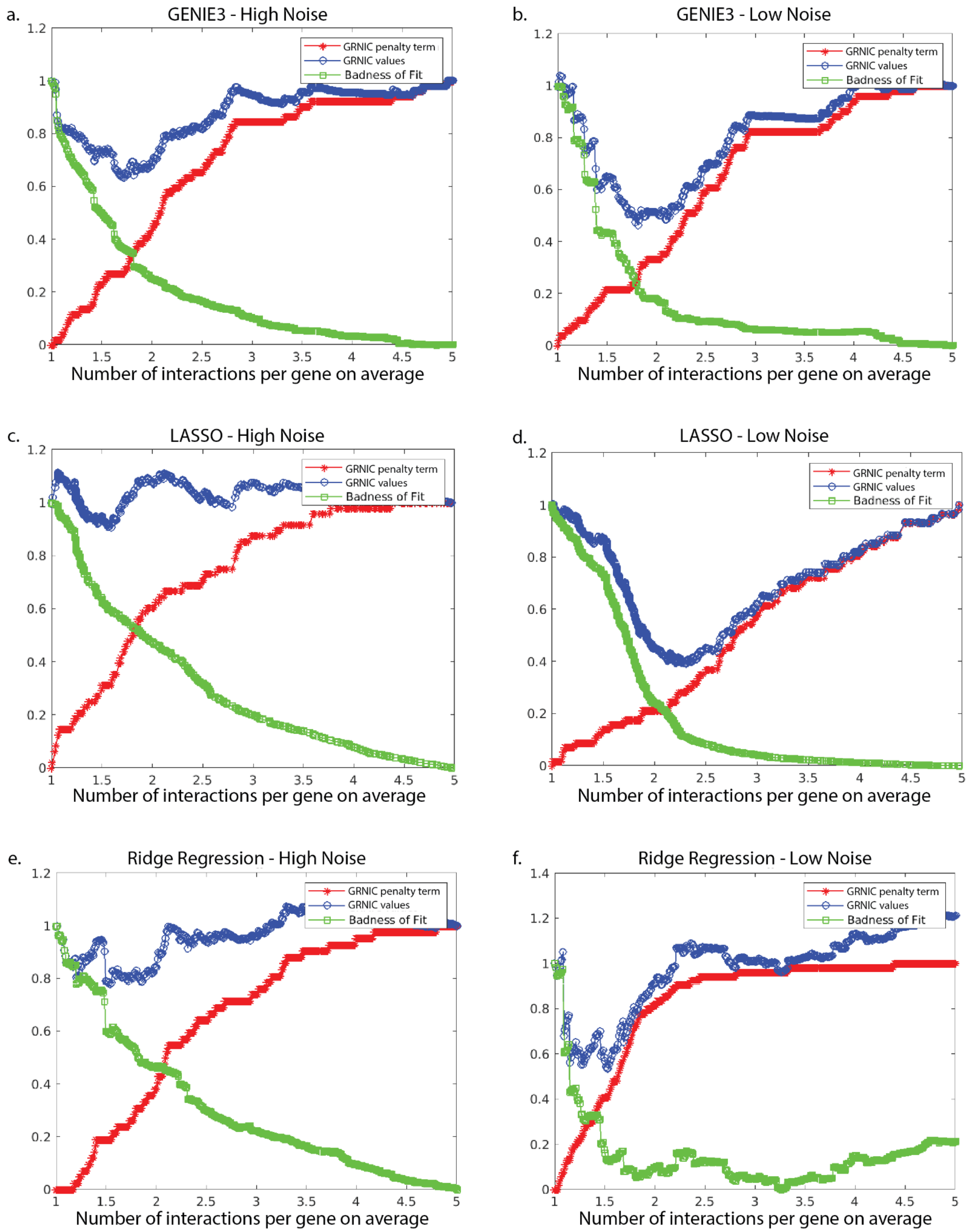

Supplementary Figure S1. Investigating GRNIC across different sparsities for inferred GRNs from network 1. ‘GRNIC values’ refers to the GRNIC model selection criterion calculated for each GRN model, inferred by (**a**-**b**) GENIE3, (**c**-**d**) LASSO, and (**e**-**f**) Ridge regression, from ‘GRNIC penalty term’ (*K*), which is the normalized number of regulators in the model, and ‘Badness of fit’ (*L*), which corresponds to the normalized prediction errors. The left panels (**a**, **c**, **e**) and the right panels (**b**, **d**, **f**) correspond to the datasets with high and low noise levels, respectively.

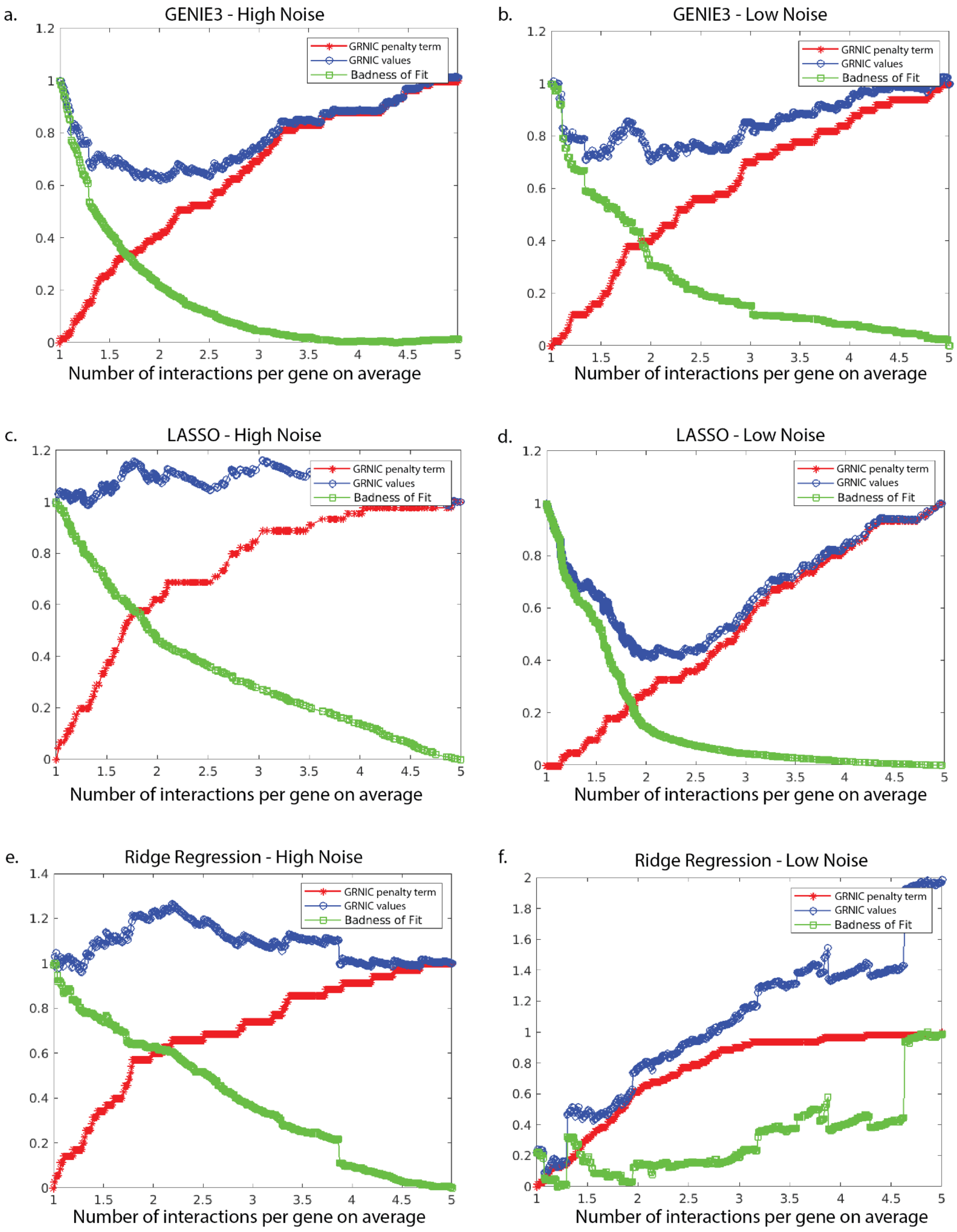

Supplementary Figure S2. Investigating GRNIC across different sparsities for inferred GRNs from network 2. ‘GRNIC values’ refers to the GRNIC model selection criterion calculated for each GRN model, inferred by (**a-b**) GENIE3, (**c-d**) LASSO, and (**e-f**) Ridge regression, from ‘GRNIC penalty term’ (K), which is the normalized number of regulators in the model, and ‘Badness of fit’ (L), which corresponds to the normalized prediction errors. The left panels (**a**, **c**, **e**) and the right panels (**b**, **d**, **f**) correspond to the datasets with high and low noise levels, respectively.

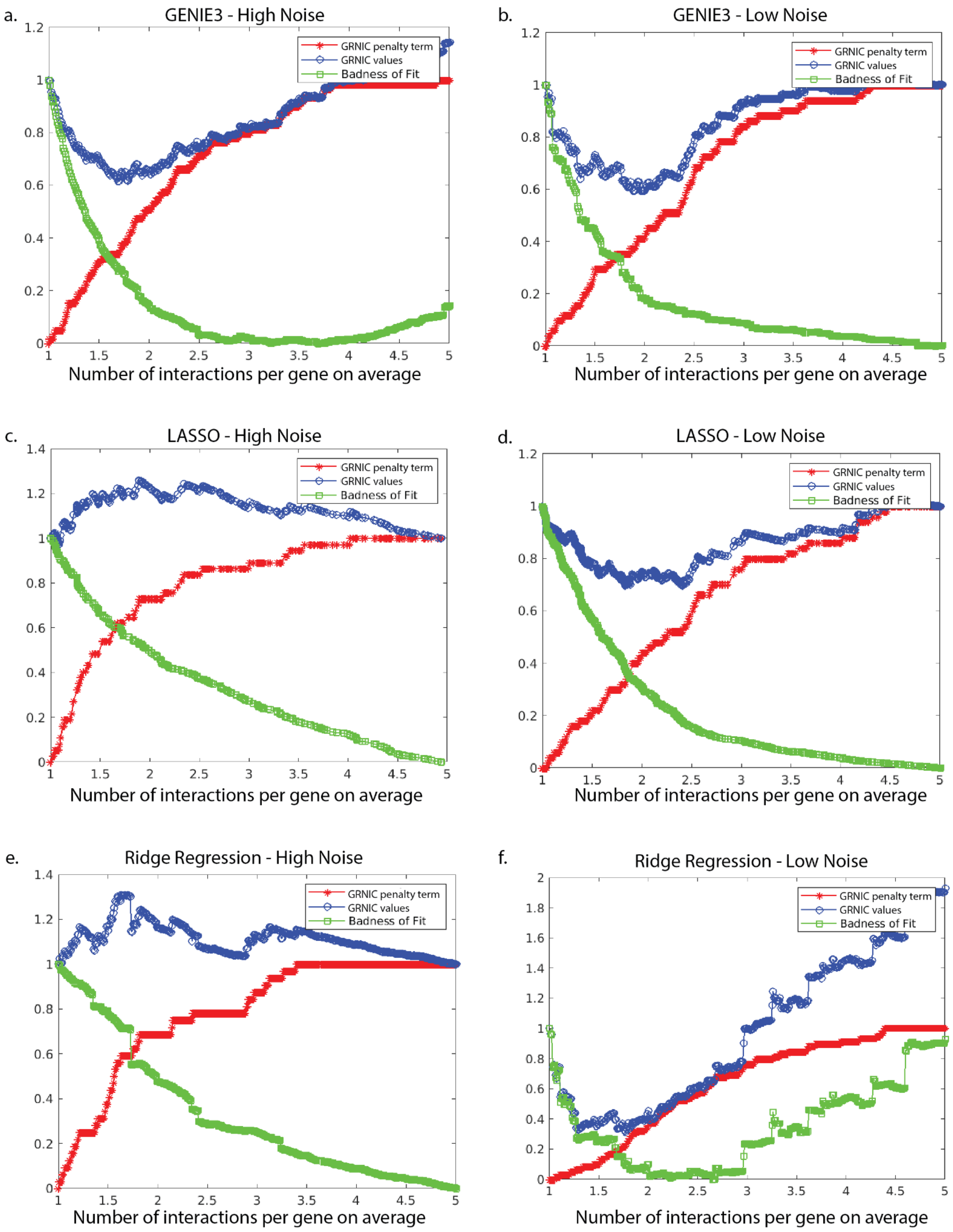

Supplementary Figure S3. Investigating GRNIC across different sparsities for inferred GRNs from network 3. 'GRNIC values' refers to the GRNIC model selection criterion calculated for each GRN model, inferred by (**a**-**b**) GENIE3, (**c**-**d**) LASSO, and (**e**-**f**) Ridge regression, from 'GRNIC penalty term' (*K*), which is the normalized number of regulators in the model, and 'Badness of fit' (*L*), which corresponds to the normalized prediction errors. The left panels (**a**, **c**, **e**) and the right panels (**b**, **d**, **f**) correspond to the datasets with high and low noise levels, respectively.

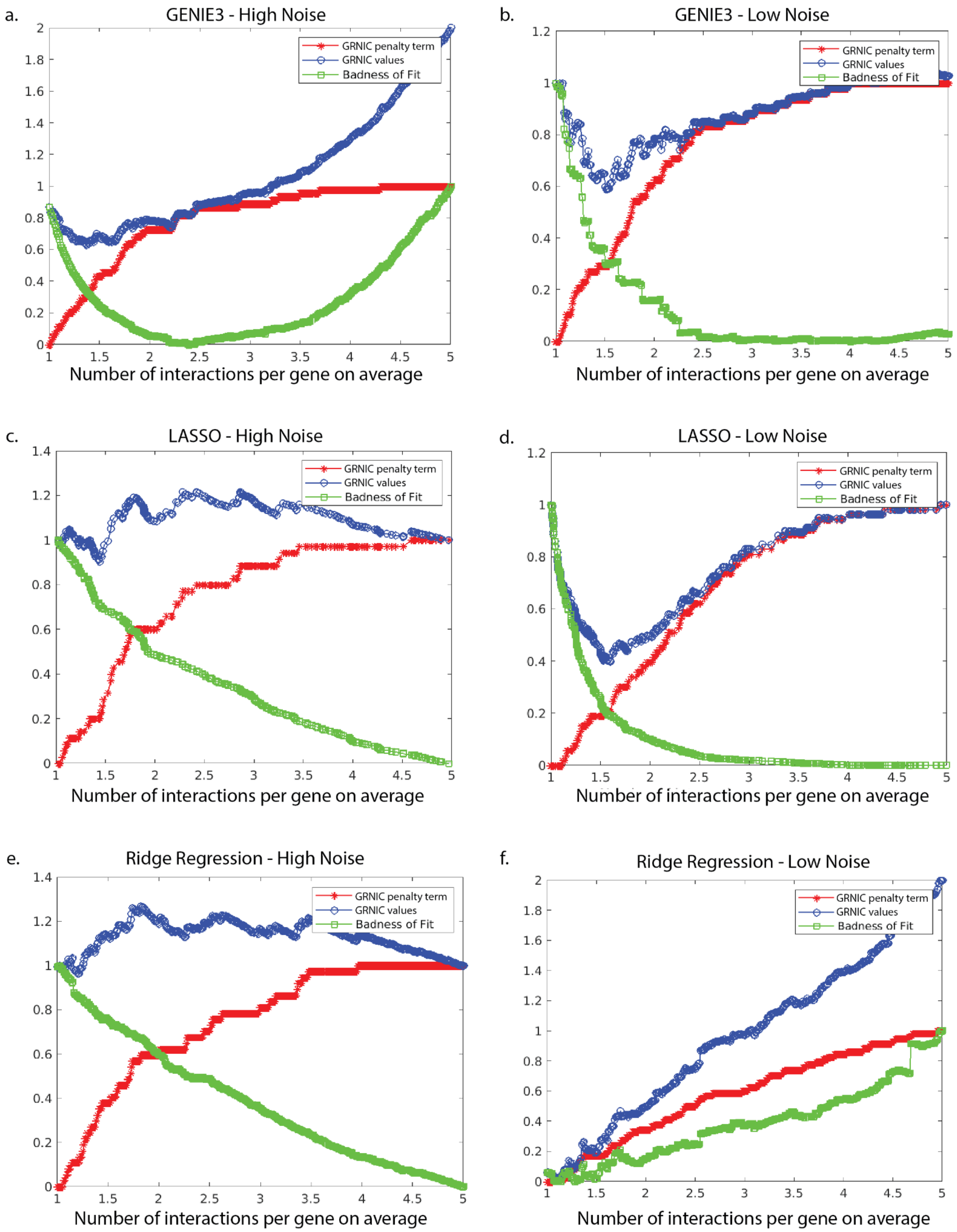

Supplementary Figure S4. Investigating GRNIC across different sparsities for inferred GRNs from network 4. 'GRNIC values' refers to the GRNIC model selection criterion calculated for each GRN model, inferred by (**a-b**) GENIE3, (**c-d**) LASSO, and (**e-f**) Ridge regression, from 'GRNIC penalty term' (*K*), which is the normalized number of regulators in the model, and 'Badness of fit' (*L*), which corresponds to the normalized prediction errors. The left panels (**a**, **c**, **e**) and the right panels (**b**, **d**, **f**) correspond to the datasets with high and low noise levels, respectively.

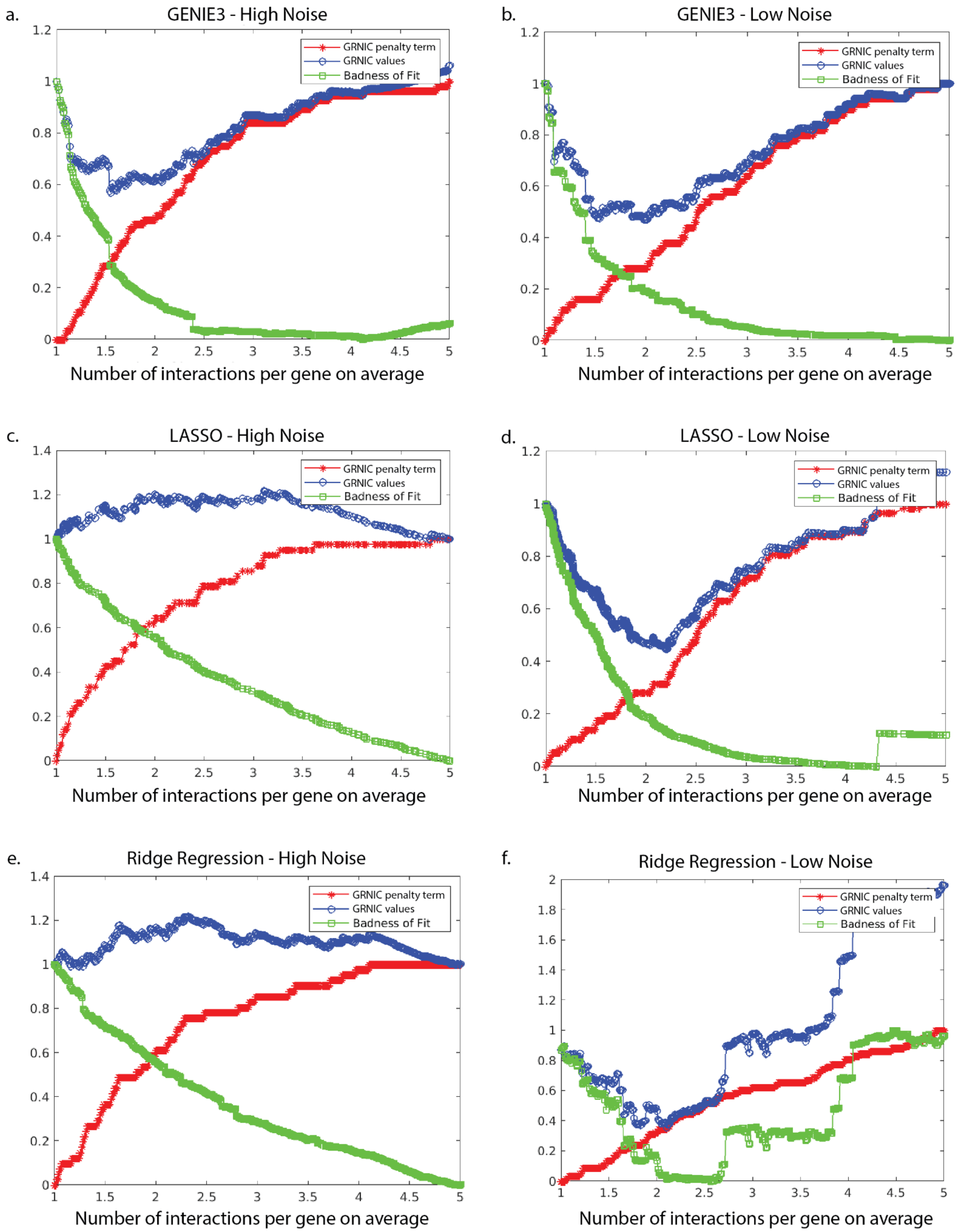

Supplementary Figure S5. Investigating GRNIC across different sparsities for inferred GRNs from network 5. 'GRNIC values' refers to the GRNIC model selection criterion calculated for each GRN model, inferred by (**a-b**) GENIE3, (**c-d**) LASSO, and (**e-f**) Ridge regression, from 'GRNIC penalty term' (*K*), which is the normalized number of regulators in the model, and 'Badness of fit' (*L*), which corresponds to the normalized prediction errors. The left panels (**a**, **c**, **e**) and the right panels (**b**, **d**, **f**) correspond to the datasets with high and low noise levels, respectively.

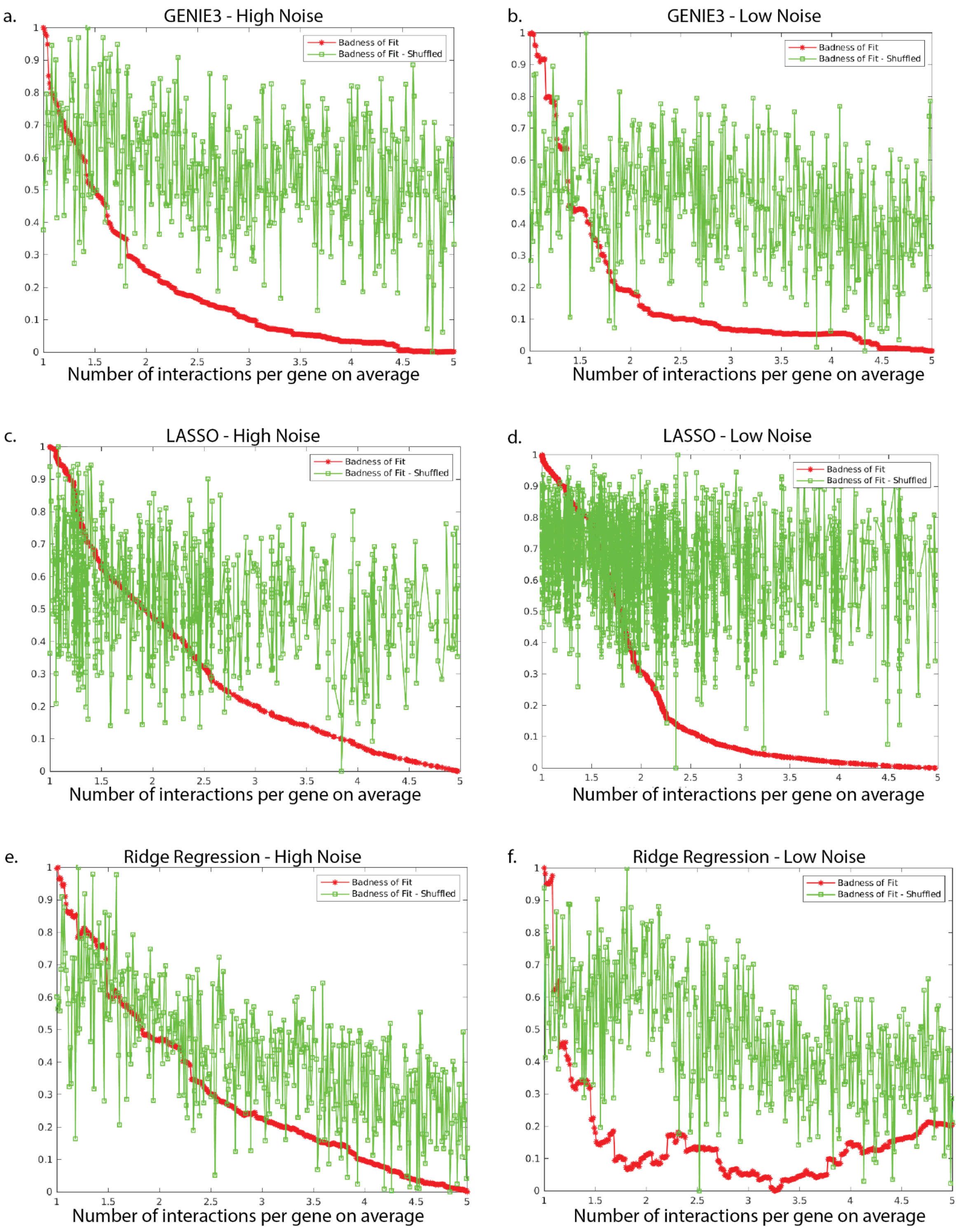

Supplementary Figure S6. Analyzing how badness of fit (*L*) is affected by randomization in datasets from network 1. 'Badness of Fit', *L*, and 'Badness of Fit - Shuffled', $L_{shuffled}$, refer to the normalized prediction errors calculated for each GRN inferred by (**a**-**b**) GENIE3, (**c**-**d**) LASSO, and (**e**-**f**) Ridge regression in terms of their ability to estimate gene expression ($\boldsymbol{Y}_{estn}$) and experiment-wise randomized estimated gene expression ($\boldsymbol{Y}_{estn\_shuffled}$), respectively. The left panels (**a**, **c**, **e**) and the right panels (**b**, **d**, **f**) correspond to the datasets with high and low noise levels, respectively.

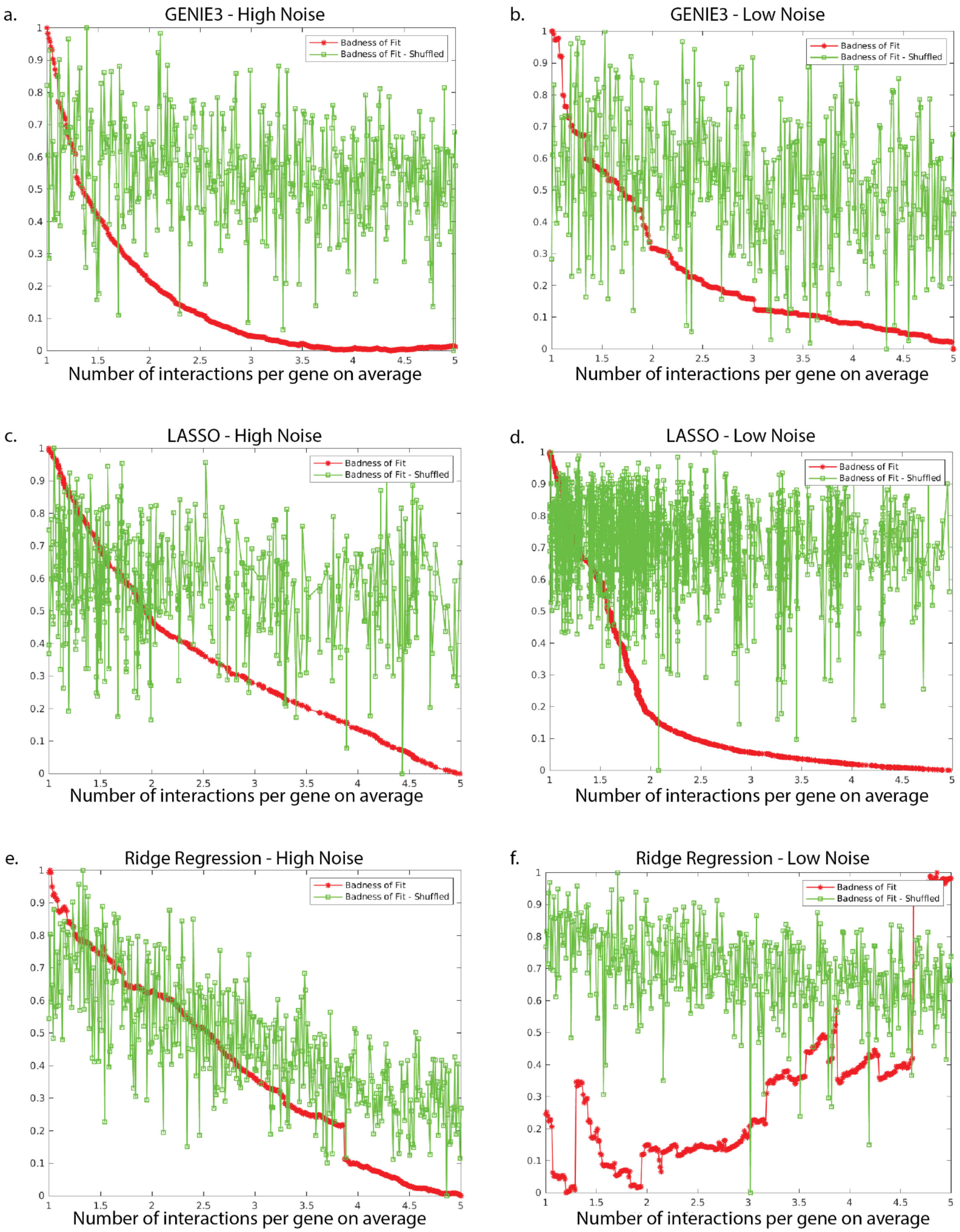

Supplementary Figure S7. Analyzing how badness of fit (L) is affected by randomization in datasets from network 2. 'Badness of Fit', L, and 'Badness of Fit - Shuffled', $L_{shuffled}$, refer to the normalized prediction errors calculated for each GRN inferred by (**a**-**b**) GENIE3, (**c**-**d**) LASSO, and (**e**-**f**) Ridge regression in terms of their ability to estimate gene expression ($\boldsymbol{Y_{estn}}$) and experiment-wise randomized estimated gene expression ($\boldsymbol{Y_{estn\_shuffled}}$), respectively. The left panels (**a**, **c**, **e**) and the right panels (**b**, **d**, **f**) correspond to the datasets with high and low noise levels, respectively.

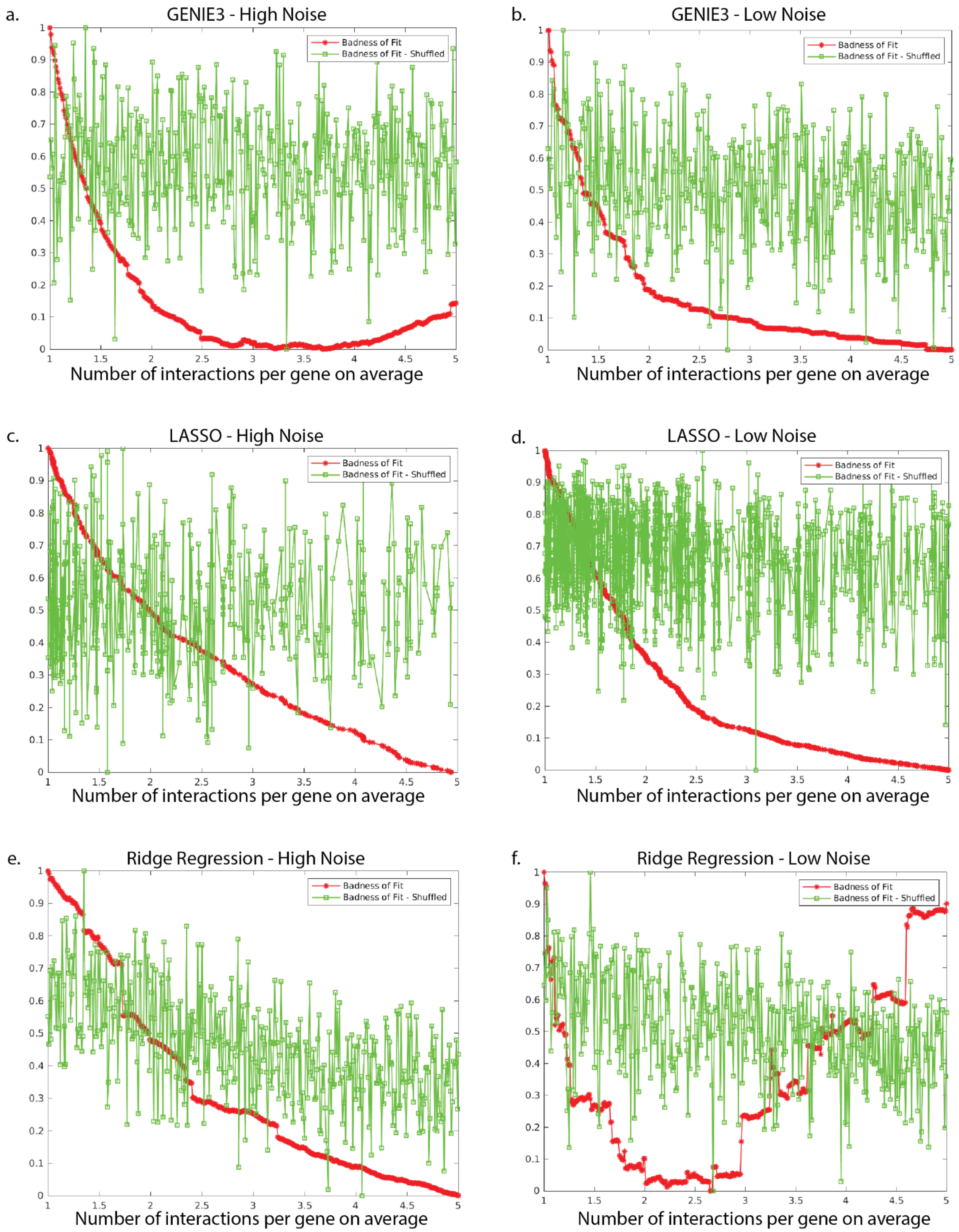

Supplementary Figure S8. Analyzing how badness of fit (L) is affected by randomization in datasets from network 3. 'Badness of Fit', L, and 'Badness of Fit - Shuffled', $L_{shuffled}$, refer to the normalized prediction errors calculated for each GRN inferred by (**a-b**) GENIE3, (**c-d**) LASSO, and (**e-f**) Ridge regression in terms of their ability to estimate gene expression ($\boldsymbol{Y}_{estn}$) and experiment-wise randomized estimated gene expression ($\boldsymbol{Y}_{estn\_shuffled}$), respectively. The left panels (**a**, **c**, **e**) and the right panels (**b**, **d**, **f**) correspond to the datasets with high and low noise levels, respectively.

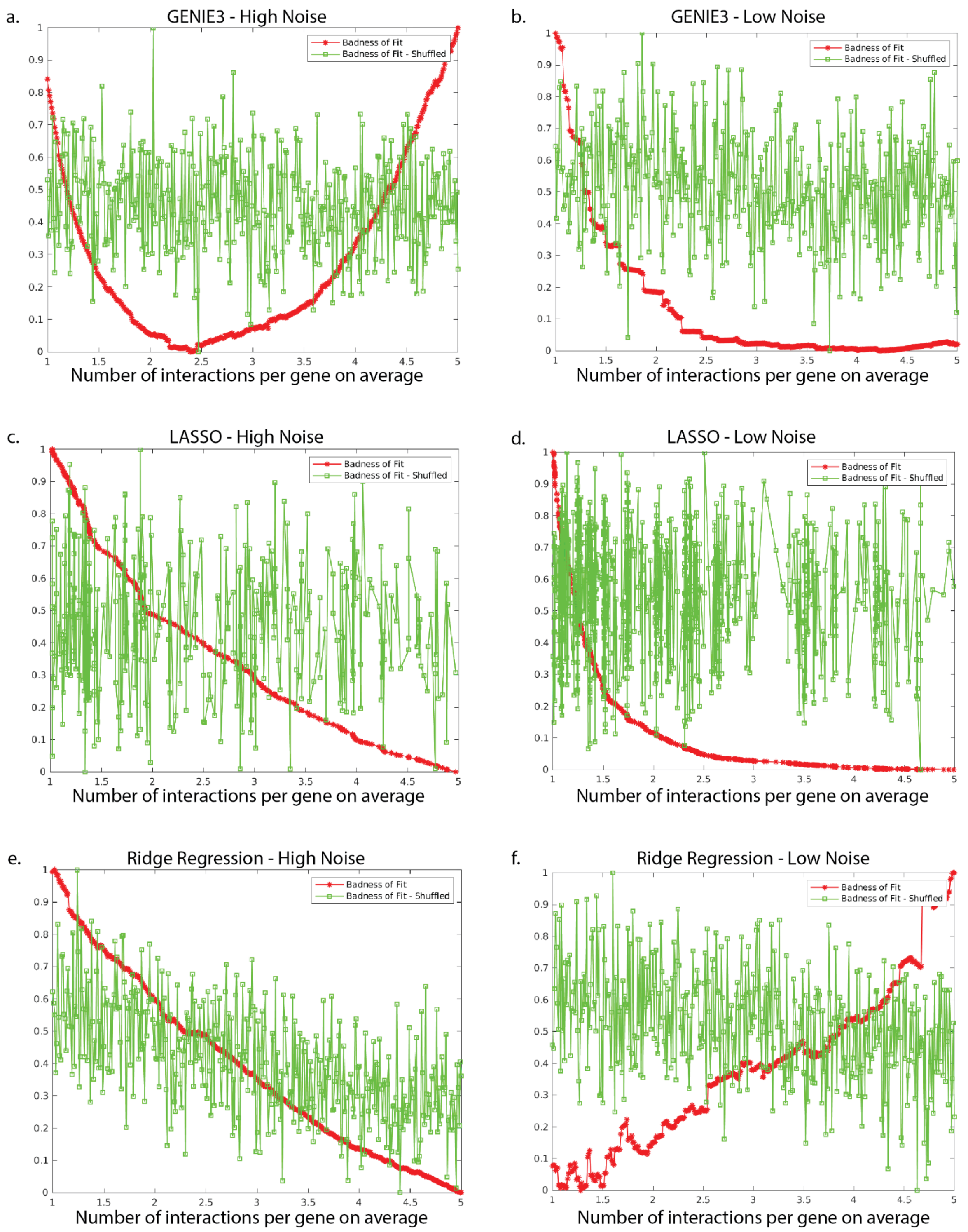

Supplementary Figure S9. Analyzing how badness of fit (L) is affected by randomization in datasets from network 4. 'Badness of Fit', L, and 'Badness of Fit - Shuffled', $L_{shuffled}$, refer to the normalized prediction errors calculated for each GRN inferred by (**a-b**) GENIE3, (**c-d**) LASSO, and (**e-f**) Ridge regression in terms of their ability to estimate gene expression ($\boldsymbol{Y}_{estn}$) and experiment-wise randomized estimated gene expression ($\boldsymbol{Y}_{estn\_shuffled}$), respectively. The left panels (**a**, **c**, **e**) and the right panels (**b**, **d**, **f**) correspond to the datasets with high and low noise levels, respectively.

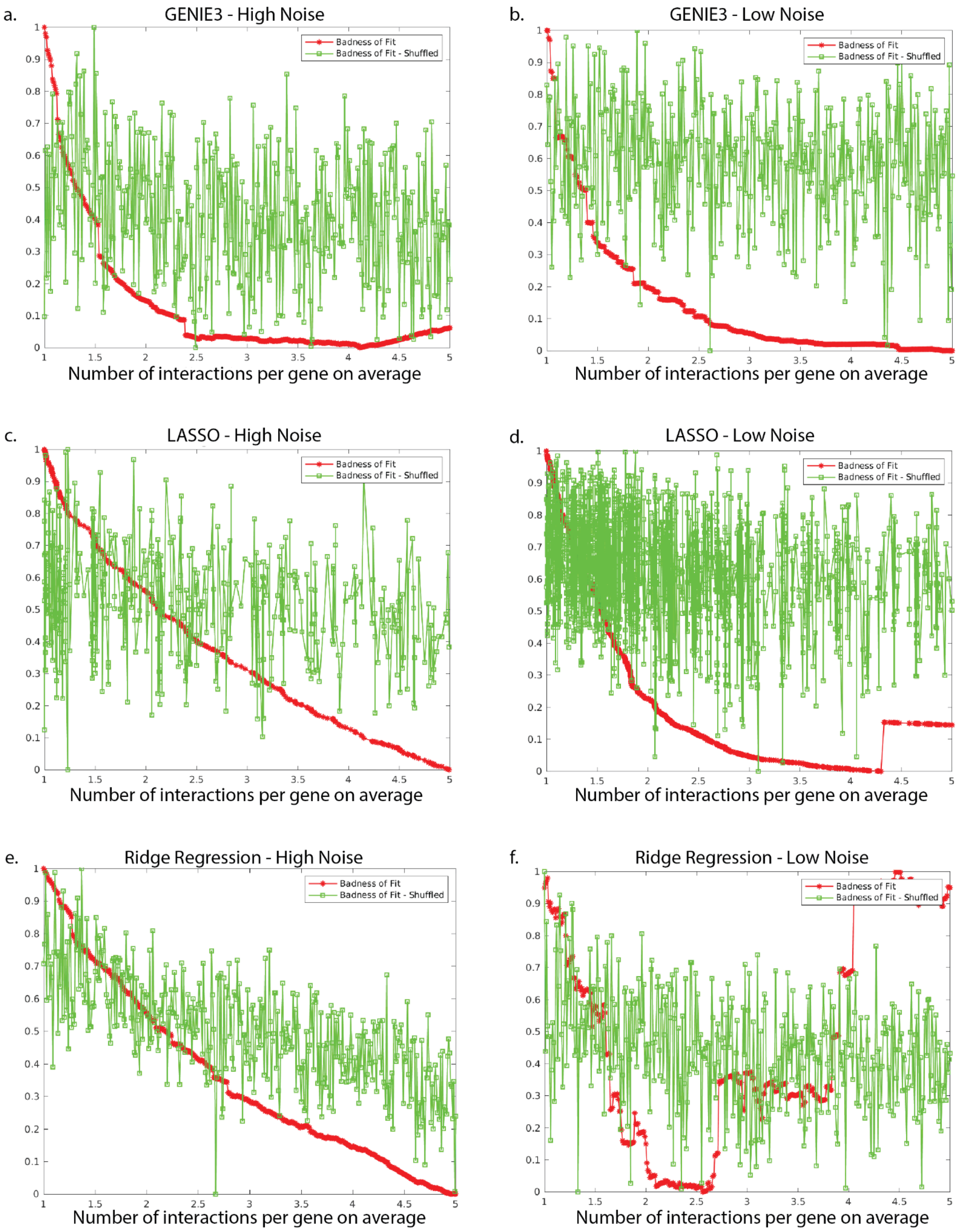

Supplementary Figure S10. Analyzing how badness of fit (L) is affected by randomization in datasets from network 5. 'Badness of Fit', L, and 'Badness of Fit - Shuffled', $L_{shuffled}$, refer to the normalized prediction errors calculated for each GRN inferred by (**a**-**b**) GENIE3, (**c**-**d**) LASSO, and (**e**-**f**) Ridge regression in terms of their ability to estimate gene expression ($\boldsymbol{Y}_{estn}$) and experiment-wise randomized estimated gene expression ($\boldsymbol{Y}_{estn\_shuffled}$), respectively. The left panels (**a**, **c**, **e**) and the right panels (**b**, **d**, **f**) correspond to the datasets with high and low noise levels, respectively.

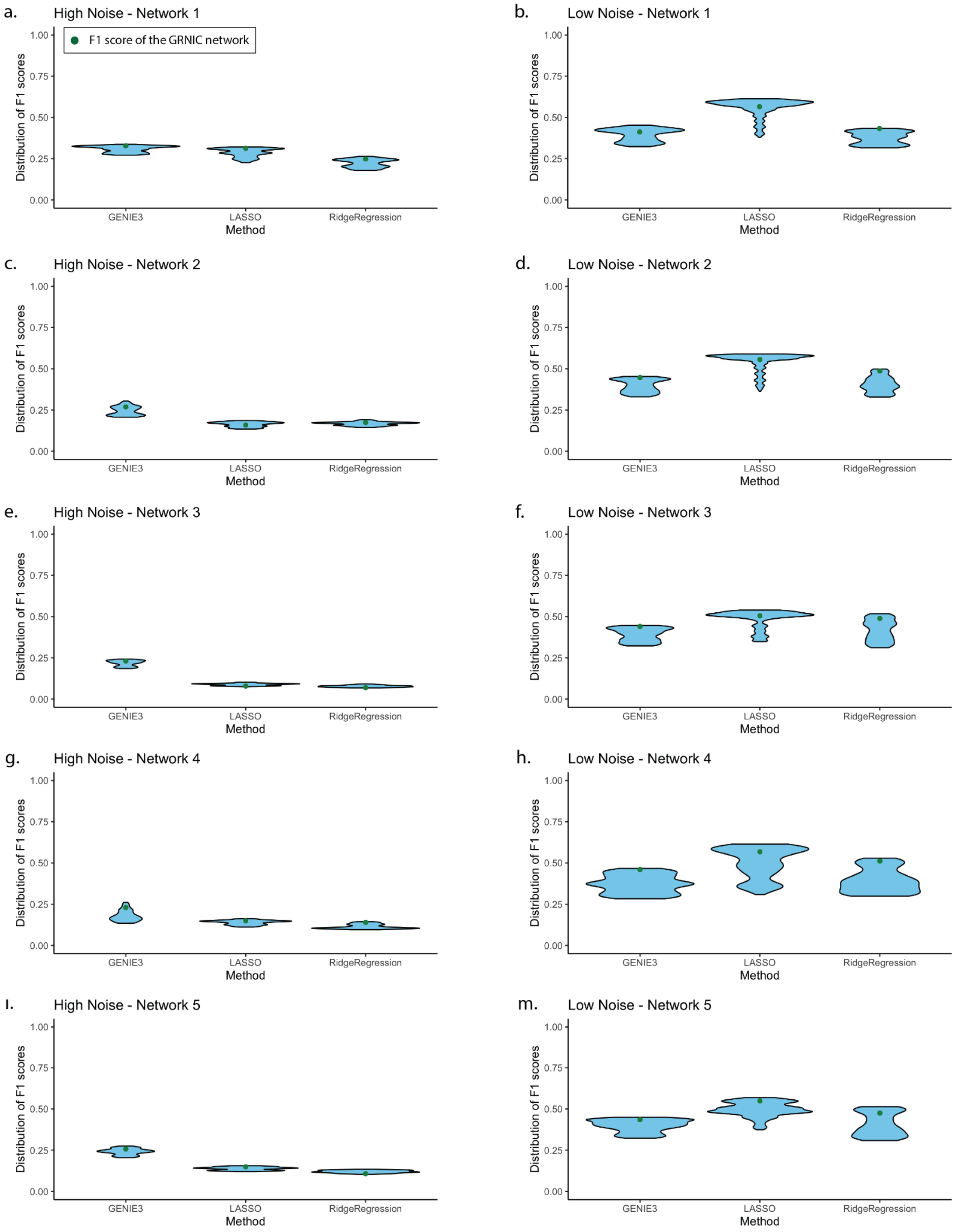

Supplementary Figure S11. Combined distribution of F1 scores across different sparsities. Blue violins represent the F1 score distributions shown (on the y-axes) per method (shown on the x-axes), and the green dots represent the F1 score of the GRN selected by SPA utilizing GRNIC. Left panels (a, c, e, g, i) and right panels (b, d, f, h, m) denote high and low noise levels, respectively. Each row on the plot represents one of five networks.

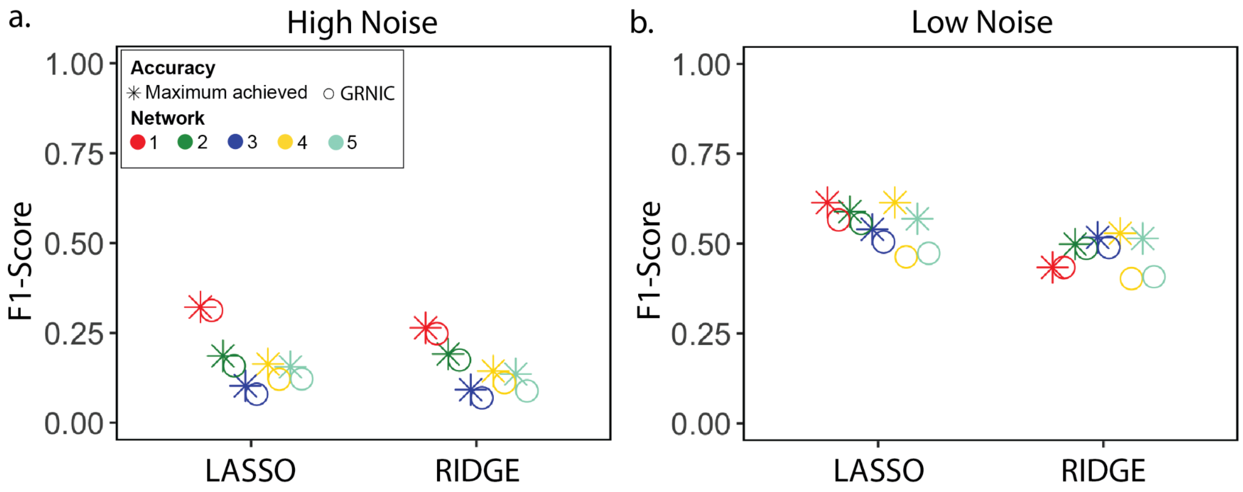

Supplementary Figure S12. Performance evaluation of the sparsity selection pipeline in terms of the F1-score when also considering selfloops. F1-scores of the inferred GRNs with selfloops from datasets generated by GeneNetWeaver with **(a)** high and **(b)** low noise levels. Each panel contains F1-scores from 5 datasets for 2 categories: GRNIC (circle), and maximum achieved in inference (star).
